# Seismic risk quantification and GIS-based seismic risk maps for Dubai-UAE_Dataset

**DOI:** 10.1016/j.dib.2021.107566

**Published:** 2021-11-12

**Authors:** Mohammad AlHamaydeh, Ghaith Al-Shamsi, Nader Aly, Tarig Ali

**Affiliations:** Department of Civil Engineering, College of Engineering, American University of Sharjah, PO Box 26666, Sharjah, United Arab Emirates

**Keywords:** Seismic resilience, Earthquake damage, Socioeconomic losses, Preparedness, GIS, Dubai

## Abstract

The data provided in this article quantifies the potential seismic losses in multi-story buildings located in Dubai, UAE. Besides, it developed GIS-based seismic risk maps, which form essential inputs towards the seismic resilience of buildings. The GIS data described herein come from different sources. The base map including the major roads, key land use classes, and administrative boundaries are from the ArcMap maps library. The Dubai districts were digitized in ArcMap from a rectified Worldview imagery of Dubai. The seismic risk analysis data are represented as attributes of the centroids of Dubai districts, which is the main data layer. The GIS-based seismic risk maps are raster GIS layers, which are created in ArcGIS using the Inverse Distance Weighted (IDW) interpolation method. The base map template is from the ArcMap maps library.

**Specifications Table**

**Table:** 

Subject	Civil and Structural Engineering
Specific subject area	Seismic risk assessment of multi-story buildings in UAE
Type of data	Table GIS File (ArcGIS/ArcMap)
How data were acquired	The seismic performance of the studied buildings was evaluated using Incremental Dynamic Analysis (IDA), which was performed using the 44 natural far-field ground motions specified in FEMA P695 report [1]. The ground motions were scaled to match the response spectra of a conservative estimate of the expected seismicity in Dubai. Fragility curves were developed for three damage states, namely Immediate Occupancy (IO), Life Safety (LS), and Collapse Prevention (CP). Socioeconomic losses were evaluated at the Maximum Considered Earthquake (MCE) based on the rates suggested in the ATC-13 [2] report and SEAOC blue book [3], respectively. Finally, corresponding seismic risk maps were generated for Dubai. In the GIS-based maps, the point data layer represents the centroids of the Dubai district layer obtained from the Dubai Municipality.
Data format	Raw analyzed Filtered The GIS data are provided in ArcGIS/ArcMap/KMZ documents format and shape file format.
Parameters for data collection	The data included in this article was collected from numerical simulations performed for five archetype buildings having number of floors varying from 2 to 16. The studied buildings were representative of the common buildings stock in Dubai.
Description of data collection	The input and output data for the seismic risk quantification was collected as sets of excel sheets. The base map including the major roads, key land use classes, and administrative boundaries are from the ArcMap maps library. The Dubai districts were digitized in ArcMap from a rectified Worldview imagery of Dubai. The seismic risk analysis data are represented as attributes of the centroids of Dubai districts, which is the main data layer. The GIS-based seismic risk maps are raster GIS layers, which are created in ArcGIS using the Inverse Distance Weighted (IDW) interpolation method. The base map template is from the ArcMap maps library.
Data source location	Institution: The American University of Sharjah (AUS) City/Town/Region: Sharjah Country: UAE
Data accessibility	Repository name: Mendeley online repository Direct URL to data: http://dx.doi.org/10.17632/shpfp7bdx7.3 AlHamaydeh, Mohammad; Al-Shamsi, Ghaith; Aly, Nader; Ali, Tarig (2021), “Seismic Risk Quantification and GIS-Based Seismic Risk Maps for Dubai-UAE_Dataset”, Mendeley Data, V3, https://doi.org/10.17632/shpfp7bdx7.3
Related research article	This data article is related to the research article: AlHamaydeh, M.; Al-Shamsi, G.; Aly, N.; Ali, T.; Geographic Information System-Based Seismic Risk Assessment for Dubai and UAE: a Step Towards Resilience and Preparedness, Practice Periodical on Structural Design and Construction, ASCE, Vol. 27, No. 1, 2022, pp. 04021069-24. https://doi.org/10.1061/(ASCE)SC.1943-5576.0000637.

**Value of the Data**

• The data provides essential inputs to the seismic risk assessment of multi-story buildings in Dubai, UAE.
• The included output data provide valuable points for researchers in the area of seismic risk assessment to start with or calibrate against.
• The data is of great benefit to researchers working in the areas of seismic risk and hazard assessment, seismic losses, and GIS-based seismic risk maps.
• The provided data might be used as a starting point to extend the evaluation of the seismic risk and losses of multi-story buildings in Dubai, UAE.
• The GIS data are essential because it provides a mapping perspective, which is useful in quantifying the seismic risk of the building stock in Dubai. This data would be of interest to researchers who are interested to examine the seismic hazard in Dubai, UAE and similar areas.
• The GIS-based seismic risk maps are very valuable in illustrating the outcome of the seismic risk assessment study and effective for evaluating seismic resilience and preparedness of the city of Dubai. As an example, the following seismic risk map in Fig. 1 below illustrates the estimated number of fatalities at the Maximum Considered Earthquake (MCE) level at the Life Safety (LS) performance level suggested by ASCE/SEI41-17 [4].

**Fig. 1:**
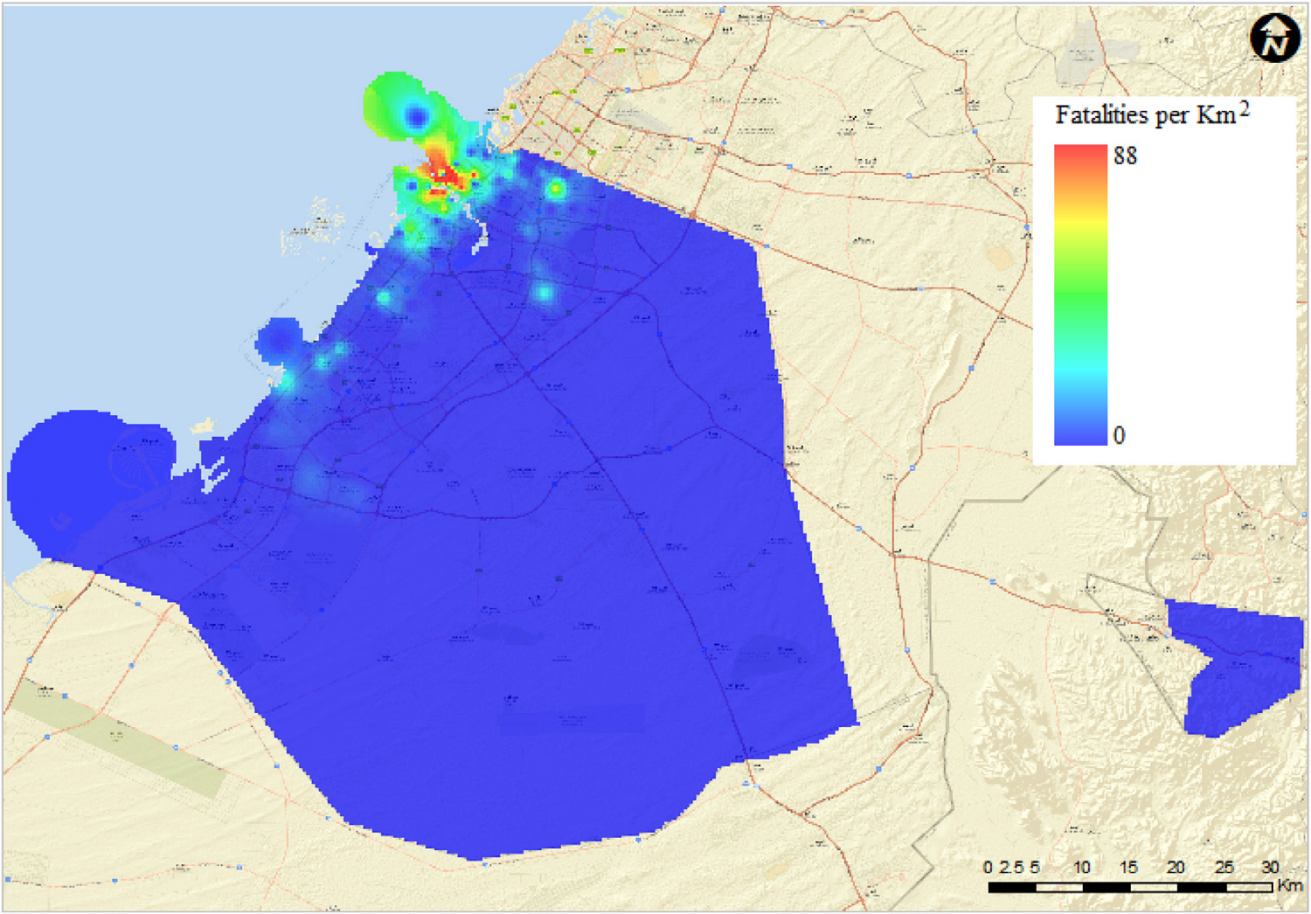
Estimated number of fatalities/km^2^ in Dubai at the Life Safety (LS) performance level.

## 1 Data Description

The data is provided in an excel file, which has three tabs. The first tab summarizes the different areas of Dubai, UAE in column B, provides the usage/classification of each area in column C, clarifies the type of assigned buildings to each area in column D, provides the population of each area in column E, and shows the estimated number of buildings corresponding to each area and population in column F.

The second tab in the data file provides the probability of exceeding the three performance levels of ASCE/SEI41-17 [4], which are Immediate Occupancy (IO), Life Safety (LS), and Collapse Prevention (CP), for the different types of multi-story buildings presented herein. The first column (column A) indicates the name of the building, the second column (column B) specifies the type of the lateral force resisting system of the building, the third column (column C) provides the number of floors, the fourth column (column D) shows the occupation type, the fifth column (column E) provides the assigned percentage of this building in each occupation, and the last three columns (columns F, G and H) provide the corresponding probability of exceedance for each performance level at the Maximum Considered Earthquake (MCE) for each studied building.

The third tab in the data file provides the estimated seismic economic and human losses for the different areas in Dubai, UAE. The first column (column B) indicates the name of the area in Dubai, UAE. The second three columns (columns C, D, and E) provide the corresponding economic losses estimated in USD/m^2^, while the next three columns (columns F, G, and H) provide the economic losses but estimated in AED/m^2^. The last 9 columns (columns I-Q) provide the estimated minor human losses, serious injuries, and fatalities due to the possible seismic events in Dubai, UAE. It should be noted that the economic and human losses are provided at the three performance levels of ASCE/SEI41-17.

The GIS data described herein come from different sources. The base map including the major roads, key land use classes, and administrative boundaries are from the ArcMap maps library. The Dubai districts were digitized in ArcMap from a rectified Worldview imagery of Dubai. The seismic risk analysis data are represented as attributes of the centroids of Dubai districts, which is the main data layer. The GIS-based seismic risk maps are raster GIS layers, which are created in ArcGIS using the Inverse Distance Weighted (IDW) interpolation method. The base map template is from the ArcMap maps library.

## 2 Experimental Design, Materials and Methods

The data provided in this article was based on the analysis performed on five reference multi-story buildings located in Dubai, UAE. The buildings represented the common stock of residential and commercial buildings. The building inventory for Dubai was completed by assigning different representative buildings to each area in Dubai, as applicable. The assignment of the buildings to each sector was based on the sector usage. To assess the vulnerability of buildings in Dubai, 2D nonlinear models of the reference structures were created using the IDARC software [5] and subjected to the scaled suite of the input ground motions. The input ground motions were the 44 far-field earthquake records provided by FEMA-P695 [1]. Afterwards, fragility curves were derived in this study using the technique proposed by FEMA-P695. The maximum interstory drift ratio was the controlling damage state measure, while the intensity measure was taken as the Peak Ground Acceleration (PGA). Finally, the limit states recommended by ASCE/SEI 41-17 were utilized herein for the derivation of the fragility curves. The performance levels suggested by ASCE/SEI 41-17 are Immediate Occupancy (IO), Life Safety (LS), and Collapse Prevention (CP).

The developed fragility curves of the representative buildings in Dubai were used to estimate the losses due to the probable seismic hazard. Earthquake loss estimation is a function of three variables: seismic hazard, inventory data for buildings and/or population density, and finally, fragility curves. In this study, losses were estimated at the MCE level (scaling factor = 1) and presented using seismic risk maps. Two types of losses were estimated, human and economic losses. Human losses are defined by the deaths and injuries of the population, while economic losses for buildings encompass costs for repair and replacement of the building inventory.

The base map including the major roads, key land use classes, and administrative boundaries are from the ArcMap maps library. The Dubai districts were digitized in ArcMap from a rectified Worldview imagery of Dubai. The seismic risk analysis data are represented as attributes of the centroids of Dubai districts, which is the main data layer. The GIS-based seismic risk maps are raster GIS layers, which are created in ArcGIS using the Inverse Distance Weighted (IDW) interpolation method. The base map template is from the ArcMap maps library. The point data layer represents the centroids of the Dubai district layer obtained from the Dubai Municipality. Further details on the objective of this study, the methodology and results are available in [6].

## Ethics Statement

This data article is in full compliance with the ethical requirements for publication in *Data in Brief.*

## CRediT authorship contribution statement

**Mohammad AlHamaydeh:** Conceptualization, Methodology, Investigation, Writing – review & editing, Supervision, Project administration. **Ghaith Al-Shamsi:** Data curation, Writing – original draft, Writing – review & editing. **Nader Aly:** Data curation, Writing – original draft, Writing – review & editing. **Tarig Ali:** Visualization, Writing – review & editing.

## Declaration of Competing Interest

The authors declare that they have no known competing financial interests or personal relationships which have or could be perceived to have influenced the work reported in this article.
